# A Finite Element Mesh Aggregating Approach to Multiple-Source Reconstruction in Bioluminescence Tomography

**DOI:** 10.1155/2011/210428

**Published:** 2011-11-14

**Authors:** Jingjing Yu, Fang Liu, L. C. Jiao, Shuyuan Yang, Xiaowei He

**Affiliations:** ^1^School of Computer Science and Technology, Xidian University, Xi'an 710071, China; ^2^Key Laboratory of Intelligent Perception and Image Understanding of Ministry of Education of China, Xidian University, Xi'an 710071, China; ^3^School of Information Sciences and Technology, Northwest University, Xi'an 710069, China

## Abstract

A finite element mesh aggregating approach is presented to reconstruct images of multiple internal bioluminescence sources. Rather than assuming independence between mesh nodes, the proposed reconstruction strategy exploits spatial structure of nodes and aggregation feature of density distribution on the finite element mesh to adaptively determine the number of sources and to improve the quality of reconstructed images. With the proposed strategy integrated in the regularization-based reconstruction process, reconstruction algorithms need no a priori knowledge of source number; even more importantly, they can automatically reconstruct multiple sources that differ greatly in density or power.

## 1. Introduction

Bioluminescence tomography (BLT) is a rapidly growing field of research in optical molecular imaging, which allows for the visualization of normal and abnormal cellular processes in living subjects at the molecular or genetic level [1–4]. With BLT, we seek to recover the spatial distribution of bioluminescent light source inside a small animal from external noninvasive measurements [5]. Generally speaking, the internal source intensity is closely related to the strength of the molecular/cellular activity, such as gene expression [6]. Thus, this imaging modality can provide in-depth information of the internal biological sources concerned in longitudinal monitoring and quantitative assessment changes and efficacy and thus further facilitates our understanding of bio-molecular processes as they occur in living animals.

When using BLT technique to measure efficiency of a genic therapy or to observe the growth or migration of cancer cells, accurate detection of different sources that differ greatly in density or power is instrumental; for example, it may yield a great deal of information regarding tumor dissemination and burden in various sites before the development of gross disease [1, 7, 8]. Therefore, the emphasis of this paper is multiple-source reconstruction that has not been sufficiently considered to date in BLT.

Most reconstruction methods for BLT can be classified to model-based reconstruction [9]. In this case, given a light propagation model, the flux on the boundary can be predicted with numerical methods such as the finite element method (FEM) by combing with the structural information and optical parameters regarding different organs. And then the BLT is formulated as an optimization problem of minimizing the discrepancy between the boundary measurements and the predicted light intensities on the tissue surface [10].

In the reconstruction procedure, the ill posedness of the BLT problem does pose a challenge for determining a unique solution of the tomographic problem. Different strategies have been proposed for coping with the ill posedness of BLT inverse problems. These studies obtain stable reconstruction by increasing the amount of independent measurements with spectrally resolved approaches [11–13], or by reducing the number of unknowns [10, 14], or with regularization techniques to incorporate some *a priori* information regarding the inverse source problem [15–17]. In this paper, we focus our attention on the multiple-source reconstruction with monochromatic boundary measurements where regularization techniques are inevitable in the reconstruction process.

The existing regularization-based reconstruction schemes in bioluminescent imaging to date can be loosely classified into three categories: *l*_2_ regularization, *l*_1_ regularization, and implicit regularization such as TSVD and LSQR [18, 19]. Through regularization, some constraints are applied to reconstruction and yield an approximate solution of the BLT problem. No matter which regularizer is used, source location and visualization are still needed for preclinical practice. Most source location schemes are directly based on the reconstructed density vector and the larger the density, the more probable the source center. Specifically, according to a priori knowledge of the number of sources, several nodes with larger density values are identified as the promising sources or set a global threshold by referring to the maximum density and only those nodes with a density value higher than the threshold will be displayed.

In most applications of BLT, for example, monitoring cancer metastasis, neither the sources number nor an appropriate global threshold is easy to determine. This is mainly due to the fact that bioluminescent lights are usually weak and diffuse, and consequently the number of potential sources is hard to estimate only by surface photon distributions. Moreover, the global threshold strategy is unfeasible for distinguishing multiple sources with distinct difference in power. Especially in *l*_2_ norm regularization cases, the obtained solution is usually oversmoothing, and thus a lower threshold will incur some artifacts in the final images whereas a higher one will discard some small potential sources. Consequently, effective reconstruction scheme for multiple sources with different powers deserves further investigation.

In this paper, we develop a finite element mesh aggregating approach for multiple-source reconstruction in BLT. The contribution of this paper to BLT reconstruction includes the following. First, we propose a multiple-source detecting strategy. Rather than assuming independence between mesh nodes, the proposed reconstruction strategy exploits spatial structure of the nodes and characteristic of energy decay to adaptively determine the number of sources and to improve the quality of reconstructed images. Second, we integrate the proposed reconstruction strategy with regularization-based inverse algorithms to build a unified framework for solving BLT inverse problem. Numerical simulations and phantom experiments demonstrate the effectiveness of this framework.

The paper is organized as follows. In Section 2, we present a multiple-source reconstruction framework with the emphasis on the finite-element-mesh-aggregating-based source detection strategy. In Section 3 we evaluate the proposed method with numerical simulations.  Section 4 presents a phantom experiment to further test the effectiveness of the proposed method. Short discussions and concluding remarks are given at the end of this paper.

## 2. Multiple-Source Reconstruction Framework

### 2.1. FEM-Based Inverse Model

Radiative transfer equation (RTE) plays an important role in image reconstruction by predicting the bioluminescence light intensities on the tissue boundary [20], but solving RTE remains an intractable task for biological tissue with spatially nonuniform optical properties and complex tissue geometries [21]. Instead, some approximations to RTE have been established to overcome the difficulty of directly solving RTE. Among them, the diffusion approximation (DA) model has been extensively used to describe the photon propagation in tissue where there is scattering dominant absorption [5–14]. Here, we restrict our discussion to the DA model for simplicity. The steady state diffusion equation complemented with the Robin boundary condition can be expressed as follows [10]:

(1) $$\begin{array}{c} -\nabla \cdot \left(D\left(r\right)\nabla \Phi \left(r\right)\right)+\mu_{a}\left(r\right)\Phi \left(r\right)=S\left(r\right),\;\left(r\in \Omega \right) \end{array},$$

(2) $$\begin{array}{c} \Phi \left(r\right)+2A\left(r;n,n^{\prime }\right)D\left(r\right)\left(v\left(r\right)\cdot \nabla \Phi \left(r\right)\right)=0,\;\left(r\in \partial \Omega \right), \end{array}$$

where Φ(**r**) is the photon power density at **r** ∈ *Ω*, *S*(**r**) is an isotropic source distribution of gene expression, and *D*(**r**) and *μ*_*a*_(**r**) are the optical diffusion and absorption coefficient, respectively. In this work, we assumed these two parameters are constant during the BLT reconstruction procedure. The term *v*(**r**) in (2) denotes the unit outer normal at boundary ∂*Ω*, *A*(*r*; *n*, *n*′) ≈ (1 + *R*(*r*))/(1 − *R*(*r*)) is the boundary mismatch factor accounting for different refractive indices across the boundary ∂*Ω*.

Following the standard finite element analysis [22], support domain *Ω* is discretized into *T* vertex nodes (*N*_1_, *N*_2_,…, *N*_*T*_) and *N*_*e*_ mesh elements, denoted as *Ω*^*l*^  (*l* = 1,2,…, *N*_*e*_); then Φ(*r*) and source term *S*(*r*) can be approximately expressed as

(3) $$\begin{array}{cc} \Phi \left(r\right)\approx \Phi^{h}\left(r\right) & =\overset{T}{\underset{k=1}{\sum }}\phi_{k}\varphi_{k}\left(r\right),\;\forall r\in \Omega ,\\ S\left(r\right)\approx S^{h}\left(r\right) & =\overset{T}{\underset{k=1}{\sum }}s_{k}\gamma_{k}\left(r\right),\;\forall r\in \Omega , \end{array}$$

where *ϕ*_*k*_ is the approximate nodal value of Φ(*r*) on the *k*th node *N*_*k*_, *φ*_*k*_(*r*) the nodal basis function with support over the elements *Ω*^*l*^, *s*_*k*_ the discretized nodal values of *S*(*r*), and *γ*_*k*_(*r*) the interpolation basis functions, which is usually the same with *φ*_*k*_(*r*).

Based on (1)–(3), a matrix equation of the linear relationship between source distribution and boundary measurements can be derived [10, Section 2]:

(4) $$AS=\Phi^{*},$$

where *A* is a typical ill-conditioned matrix and Φ* represents measurable boundary nodal photon density. In real BLT experiments, Φ* is computed from the surface flux image captured with a CCD camera.

### 2.2. General *l*_*p*_-Norm-Based Regularization

As mentioned in Section 1, the flux density on the boundary can be predicted according to a forward model, thereby a natural choice for source reconstruction is to minimize the misfit between predicted data and measurements, that is,

(5) $$S=\arg\;\underset{S}{\min}{||AS-\Phi^{*}||}^{2}.$$

To deal with the ill posedness of BLT inverse problem, permissible source region is usually incorporated into the reconstruction model by spatially constraining the reconstruction domain to the area of interest [10, 14, 16, 23]. A more effective approach to reconstruction is using regularization to act as an algebraic stabilizer in estimating solutions.

Using a general *l*_*p*_  (0 < *p* ⩽ 2) norm constraint, we reformulate the objective function for BLT reconstruction in (5):

(6) $$S_{\text{reg}}=\arg\;\underset{S}{\min}\left\{{||AS-\Phi^{*}||}_{2}^{2}+\lambda {||S||}_{p}\right\},$$

where the first term represents reconstruction error and the second is regularization term that fuses* a priori *knowledge or constrains into reconstruction. Regularization parameter *λ* > 0 provides a tradeoff between data fitting and constraints regarding solutions. Obviously, Tikhonov regularization method is a special case of (6) for *p* = 2, that is, using an *l*_2_-norm regularizer. For *p* = 1, *l*_1_-norm-based sparse regularization methods have recently attracted considerable amount of attention in BLT [17, 23–25] and the reconstructions results therein witnessed some improvements in image quality.

### 2.3. Multiple-Source Detection Strategy

Based on the solution (a source density vector) obtained in Section 2.2, source localization and imaging is then performed by combining with FEM mesh information. Facing the dilemma of threshold choice mentioned in Section 1, we are hoping for an adaptive method that can avoid the difficulty of threshold selection while at the same time removing artifacts in the reconstructed images with relatively lower computational cost.

 Consider that in most applications of BLT, for example, detecting events that occur during the early stages of disease progression, the bioluminescent sources we want to recover are often localized in some small subregions of the domain. On the other hand, because light intensity is heavily attenuated in biological tissue and falls off exponentially from the illumination point, the diffusion range of a bioluminescent source is limited by the source strength. Consequently, when taking the spatial structure of the mesh nodes into account, the source density vector should have a spatial aggregation on the mesh, which is also illustrated in the experiments in Section 3 (Figure 4). It is found that, in a very small local region, if a node in the mesh has a maximum density value, with a very high probability its adjacent nodes are also with larger density. It is found that in a very small local region, if a node in the mesh has a maximum density value, with a very high probability its adjacent nodes are also with a larger density. We also observe that there are some nodes with smaller density in the vicinity of nodes with the larger density. These observations are helpful for discriminating pseudosource from a cluster of mesh nodes and removing artifacts in images. On the basis of the above analysis, an iterative multiple-source detection strategy (MSDS) is proposed in the following steps.

**Step 1**

Obtain the regularized solution (the source density vector *S*).

**Step 2**

Threshold preprocessing. In the presence of inevitable noise, the solutions usually have many very small nonzero components. Consequently, the preprocessing of solution with a small threshold of *c*max⁡ (*S*_*i*_) is helpful to remove pseudosources and reduce the data size to be processed in the subsequent steps. For all the experiments in Section 3, the constant *c* = 0.05.

**Step 3**

Define a set *O* = {*S*_*i*_ | *i* ∈ *N*, *S*_*i*_ > 0}.

**Step 4**

Initial the sources number *k* = 1.

**Step 5**

Compute the node index *j* = arg max⁡_*S*_*i*_∈*O*_⁡(*S*_*i*_). We move the element *S*_*j*_ to a new set *P*_*k*_. By traversing set *O* we can find out the other elements that directly adjoin the node *j*, if any, according to the mesh structure information. Re-move these elements to *P*_*k*_.

**Step 6**

If set *O* is null, stop; otherwise *k* : = *k* + 1, and go to Step 5.

With the steps defined above, we provide an automatic method to estimate the number of sources from the reconstruction results iteratively. The final results contain *k* sources. Here, *k* is the number of subsets of the initial set *O* obtained at the end of the above iteration. Each subset corresponds to a reconstructed source. When *P*_*i*_  (*i* = 1, *L*, *k*) has more than one member, we call this situation “overrepresentation,” the nodes related to these elements will aggregate to represent a single source and the node with largest density value *S*_*j*_ is regarded as the source center for simplicity. Eventually, the cartesian coordinates of the reconstructed sources are obtained by their node index in the finite element mesh.

### 2.4. Regularization Framework for Multiple-Source Reconstruction

**Figure 1:**
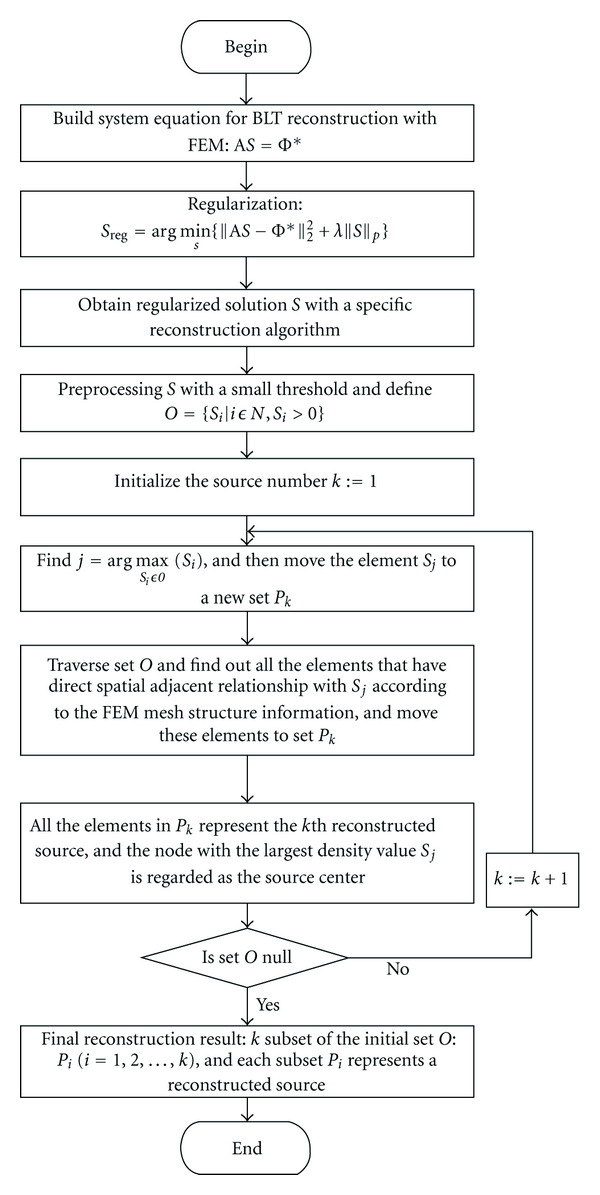
Flow chart of the regularization framework for multiple-source reconstruction.

Based on the foregoing reconstruction scheme, we build a unified regularization framework for multiple-source reconstruction by integrating the MSDS with the general *l*_*p*_-norm regularization, as shown in Figure 1.

An appealing property of this framework is its flexibility. The MSDS is a relatively independent component of the framework, and hence different regularizer and different reconstruction algorithms can be utilized according to the practice of BLT.

## 3. Numerical Results and Analysis

In this section, we present some numerical experiments to demonstrate the utility and the effectiveness of the proposed method in multiple-source settings. Comparison is performed between the proposed MSDS and the traditional global threshold strategy (GTS). It should be pointed that the main theme of this paper is to evaluate the performance of this framework for multiple-source reconstruction in BLT, rather than the comparison between specific reconstruction algorithms. As representatives of algorithms using *l*_1_ and *l*_2_ regularization, Tikhonov regularization method [26] and *l*1–*ls* [27] are, respectively, combined with the above two strategies to recover the interior source distribution from the synthetically boundary measurements. Consequently, the reconstruction methods evaluated in the following experiments include Tikhonov + MSDS, Tikhonov + GTS, *l*1–*ls* + MSDS, and *l*1–*ls* + GTS.

It is known that regularization parameter is crucial to yield a good solution for ill-posed problems, and the choice of regularization parameter is usually nontrivial. In this paper, the regularization parameter for Tikhonov method was determined with the adaptive method proposed in [28]. As for *l*1–*ls*, the parameter *λ* was chosen as suggested in [27], that is, *λ* = 0.1||2*A*^*T*^Φ*||_*∞*_.

**Figure 2:**
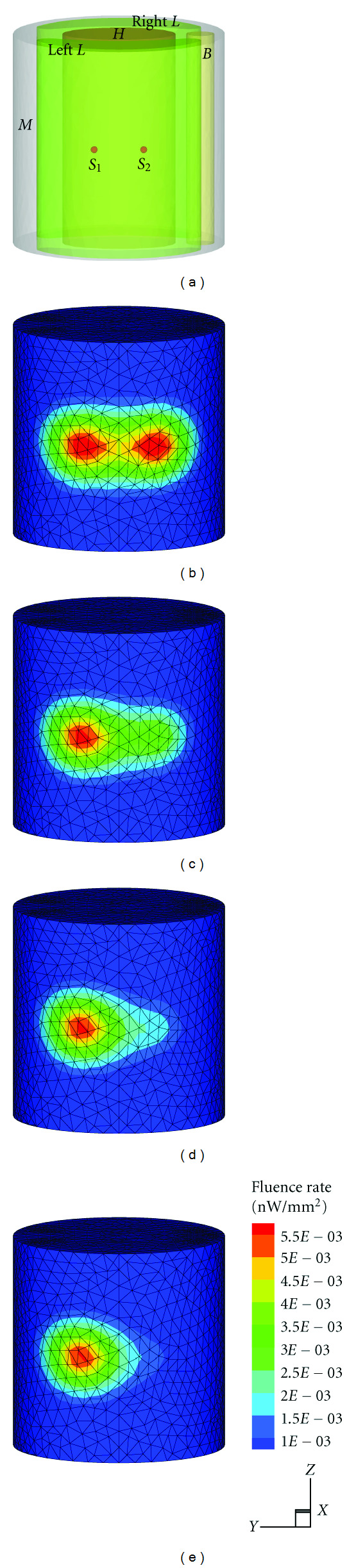
(a) 3D view of the heterogeneous phantom with two sphere sources in the left lung. (b)–(e) Different photon distributions generated, respectively, in power ratio of 1 : 1, 2 : 1, 4 : 1, and 8 : 1 cases.

All the experiments were performed on a cylindrical mouse chest numerical phantom as shown in Figure 2(a). The heterogeneous model is 30 mm in diameter and 30 mm high. The specific optical properties of different organs are listed in Table 1 [14].

### 3.1. Reconstruction for Double Sources with Different Powers

In the first study, we consider the ability to resolve sources with different powers. Two sphere sources with radius of 0.5 mm were positioned in the left lung with the centers at *S*_1_ = (−9, −3.5,15) and *S*_2_ = (−9,3.5,15), respectively. They were uniform in size and shape. To illustrate the point of our discussion, we consider four cases of experiment settings: (I) both of the initial source densities were 1 nW/mm^3^; (II) to (IV) the densities of *S*_1_ were still 1 nW/mm^3^, but the densities of *S*_2_ were 0.5 nW/mm^3^, 0.25 nW/mm^3^, and 0.125 nW/mm^3^, respectively, that is, the ratios of the power of source *S*_2_ to that of *S*_1_ were 2 : 1, 4 : 1, and 8 : 1.

In the following experiments, the model was discretized into a fine tetrahedral element mesh and synthetic measurements were generated by solving the forward model with FEM. To simulate the noise involved in real BLT experiment, 10% Gaussian white noise was added to synthetic data. Figures 2(b)–2(e) show the forward mesh and the simulated photon distribution on the surface in the above four source settings. Obviously, it is difficult to predict the source number only according to the photon distribution especially in case (III) and case (IV).

In the reconstruction process, a permissible source region strategy was also employed as *a priori *information to decrease the ill posedness of BLT inverse problem, which was defined as {(*x*, *y*, *z*) | 8 < (*x*^2^ + *y*^2^)^1/2^ < 12,13.5 < *z* < 16.5} [14]. Following the proposed reconstruction framework the reconstructions were carried out with the aforementioned four methods under different source settings.

**Figure 3:**
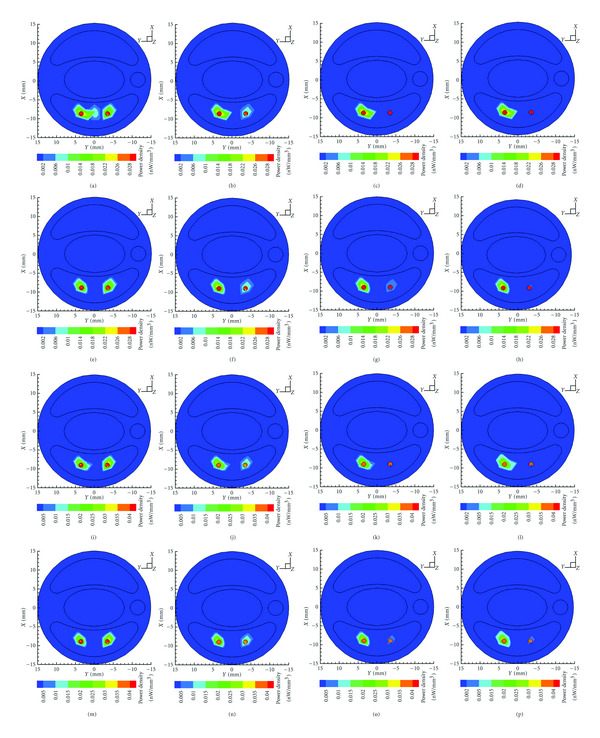
From left to right: transverse views of the reconstruction results at *z* = 15 mm in power ratio of 1 : 1, 2 : 1, 4 : 1, and 8 : 1. From top to bottom: final results of Tikhonov + GTS, Tikhonov + MSDS, *l*1–*ls* + GTS, and *l*1–*ls* + MSDS, respectively.

The first row and the third row of Figure 3 show the final reconstruction results by Tikhonov method and *l*1–*ls* method combined with the proposed MSDS. For comparison, the second row and the fourth row of Figure 3 present the corresponding reconstructed results rendered from GTS, where a global threshold (35% of the maximum density value) was used. It is obvious that the two sources are accurately detected by the proposed MSDS combined with different regularization methods in all the cases considered. On the other hand, for case (III) and case (IV), only the source with larger power is detected by Tikhonov + GTS and *l*1–*ls* + GTS, whereas the other weaker one is lost in the final reconstruction results.

To quantitativly assess reconstruction results in different power settings, we summarize location errors and reconstructed powers by different reconstruction schemes in Table 2, where the second column represents the actual initial power ratio of *S*_1_ to *S*_2_, and *S*_1_^*R*^ and *S*_2_^*R*^ denote the corresponding reconstructed sources. N/A denotes that location information is not available.

From Table 2, it is seen that *l*_1_-norm-based method *l*1–*ls* generally performs better than *l*_2_-norm-based Tikhonov method in terms of reconstructed powers and locations.

**Figure 4:**
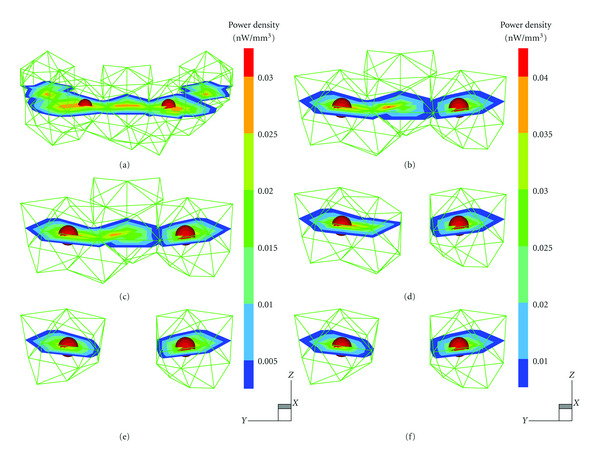
Top row: regularized solutions by Tikhonov regularization (left) and *l*1–*ls* method (right). Middle row: corresponding final reconstruction results by GTS with a threshold of 0.35max⁡ (*S*_*i*_). Bottom row: final reconstruction results by Tikhonov + MSDS (left) and *l*1–*ls* + MSDS (right).

Figure 4 illustrates the mesh aggregating process of MSDS and compares the final reconstruction results of MSDS with those of GTS in case (I). We can observe that there are some nodes with smaller density value in the vicinity of the two nodes with larger density, as shown in Figures  4(a) and 4(b). Apparently, retaining all of the nonzero components of the regularized solution will incur some artifacts in the final reconstruction image, in particular for *l*_2_ norm solution by Tikhonov regularization method. The results in Figures  4(c) and 4(d) show that the traditional GTS directly discards those nodes with density value lower than the given threshold in the final results to improve the image quality. Usually, a higher threshold is preferred in the literature, thus a threshold of 0.35max⁡ (*S*_*i*_) was used in the experiments for GTS method [16, 29]. As a result, those suspect targets with density lower than threshold will be omitted in this way. Unlike traditional methods, the proposed MSDS considers not only density value of a node but also mesh structure used in reconstruction and thus it has an ability to remove pseudosources and retain weak suspect sources in the final reconstruction results, as shown in Figures  4(e)-4(f) and 3.

### 3.2. Four-Source Reconstruction

**Figure 5:**
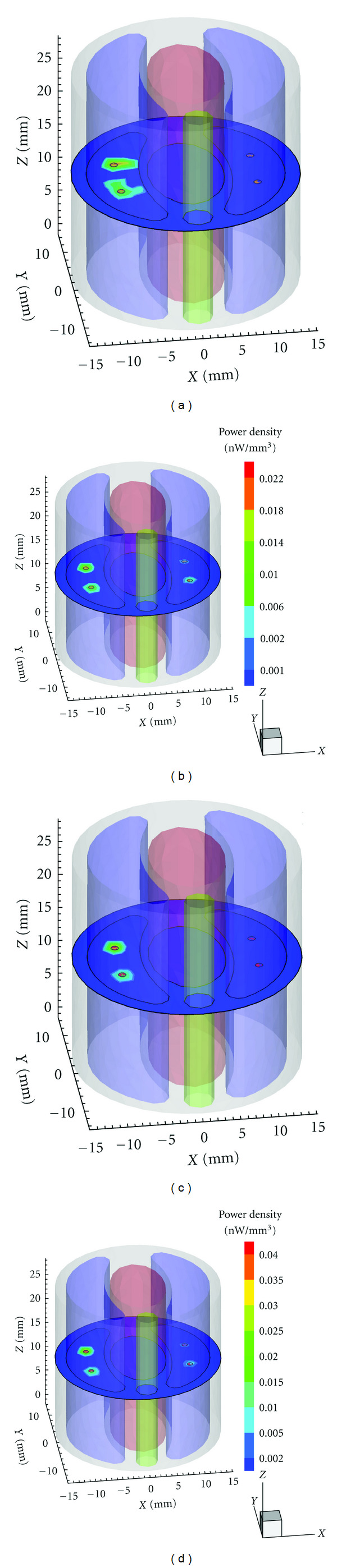
3D views of reconstruction results with synthetic data generated from four scattered sources with different powers. (a)–(d) are the results of Tikhonov + GTS, *l*1–*ls* + GTS, Tikhonov + MSDS, and *l*1–*ls* + MSDS, respectively.

In the second experiment, we attempt to reconstruct sources with synthetic data generated from four scattered sources with different initial powers, which may be a common case in tumor metastasis. Specifically, the power setup was according to ratio of 8 : 4 : 2 : 1 and the maximum power density was 1 nW/mm^3^.  Figure 5 shows 3D views of the results of Tikhonov regularization method and *l*1–*ls* method, respectively, combined with GTS and MSDS. The global threshold was the same as previous simulations. Obviously, it is hard for traditional GTS method to detect multiple sources with lower power density in such experimental setting, whereas the proposed MSDS accurately distinguishes all of the sources.

### 3.3. Influence of Finite Element Mesh

**Figure 6:**
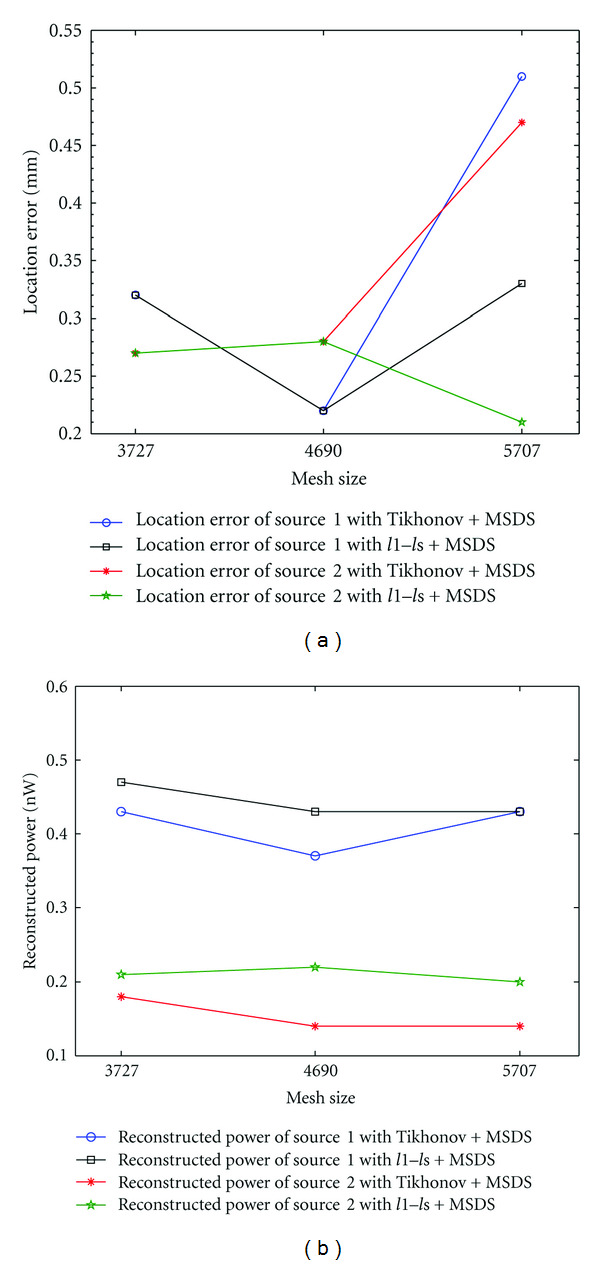
(a) Location error under different mesh levels. (b) Reconstructed power under different mesh levels.

In view of the idea that the proposed multiple-source reconstruction approach utilizes underlying mesh structure information, it is necessary to assess the influence of different FEM discretization on the proposed method. Therefore, we conducted a set of double-source experiments under different discretization level. The results in Figure 6 (where the number of nodes in reconstruction domain denotes different discretization level or mesh size) show the influence of finite element mesh on reconstruction. For Tikhonov regularization method combined with MSDS, the location error increases slightly after a decrease along with the increasing of mesh size and the reconstructed power presents a similar variation trend. As for *l*1–*ls* combined with MSDS, both location error and reconstructed power vary slightly with mesh changes.

In general, finite element discretization does affect reconstructed results in the sense that the location error and the reconstructed power vary with the change of mesh. However, for all of the discretization levels considered, the proposed method is able to accurately localize and quantify light source distribution. These results demonstrate the robustness of the proposed reconstruction framework against mesh discretization.

## 4. Phantom Study

We further demonstrate the effectiveness of the proposed reconstruction algorithm with phantom experiments. This set of BLT experiments were conducted with a dual-modality BLT/micro-CT system [17, 30]. A backthinned, backilluminated cooled CCD camera is used to measure the signal on the phantom surface from four directions at 90-degree intervals.

The heterogeneous mouse chest phantom with 30 mm height and 15 mm diameter consists of four parts that represent muscle, lungs, heart, and bone, respectively [30]. The optical properties of different organs are listed in Table 1. Two small holes of diameter 2 mm were drilled in the phantom to place glass capillary with 1 mm inside diameter. Luminescent solutions of height 2 mm were extracted from a red luminescent light stick (Glow products, Canada) and then injected to glass capillary to serve as one testing source. The generated luminescent light had an emission peak wavelength of about 650 nm. The real center positions of the two testing sources were (−9,2, 16.6) and (−9, −3,16.6).

**Figure 7:**
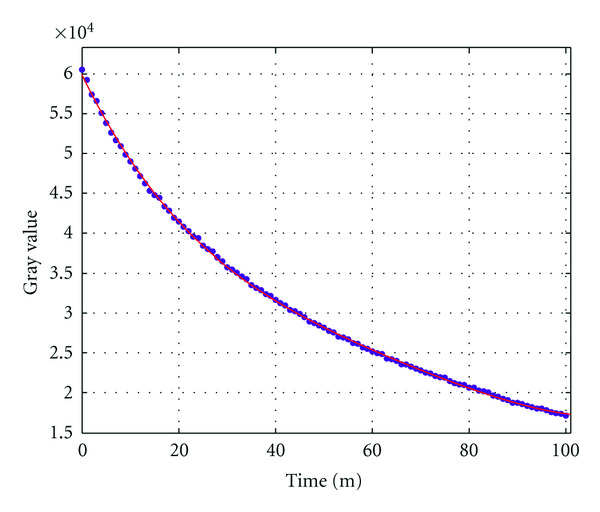
Decay curve of light density.

**Figure 8:**
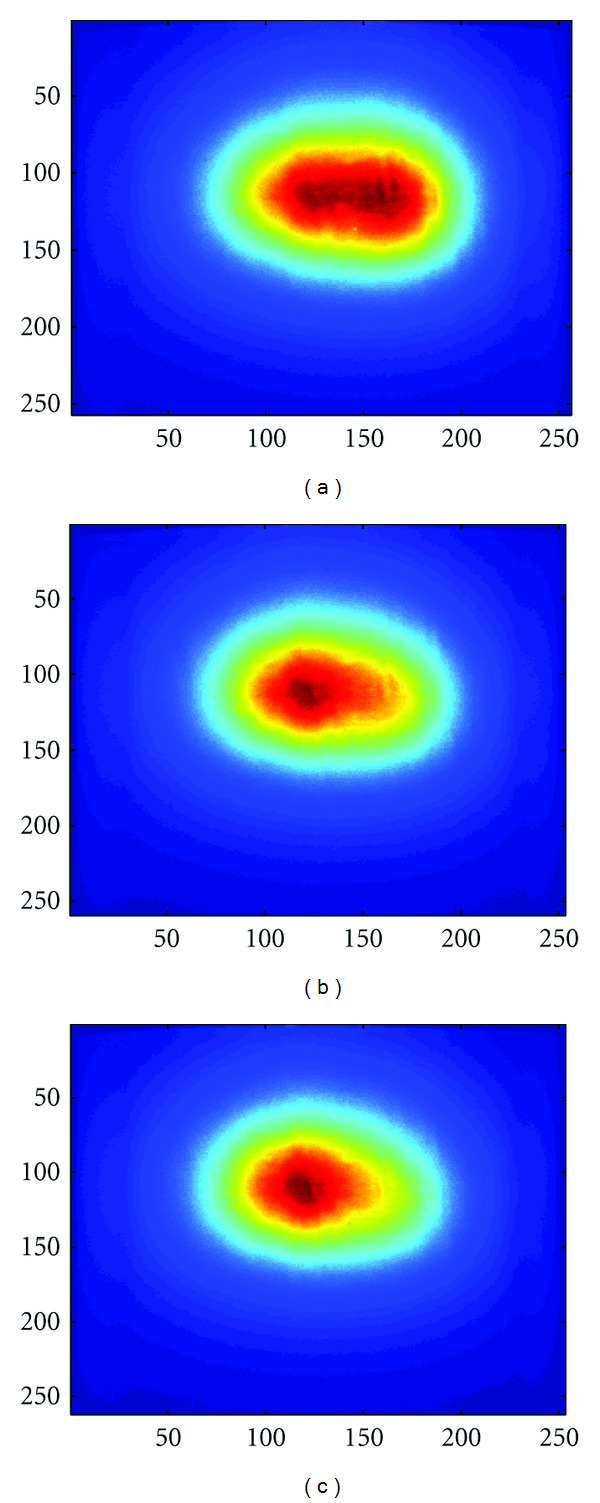
(a)–(c) Front views of measurements by CCD for the case of intensity ratios 1 : 1, 2 : 1, and 4 : 1.

It is known that luminescent light intensity will decrease with the passage of time. We collected 100 gray level images of the sources, which were taken by the CCD camera every one minute.  Figure 7 shows the fitted decay curve of light density. According to the decay curve, we can obtain sources with different intensities by controlling the injection time of luminescent solutions. Three groups of experiments were conducted, and the ratios of the intensity of source *S*_2_ to that of *S*_1_ were 1 : 1, 2 : 1, and 4 : 1, respectively. Figures 8(a)–8(c) show the front views of the corresponding measured data on CCD under different intensity settings. Subsequently, a permissible source region was roughly determined according to the surface flux density distribution, which is expressed as {(*x*, *y*, *z*) | 8 < (*x*^2^ + *y*^2^)^1/2^ < 13,15 < *z* < 18}.

**Figure 9:**
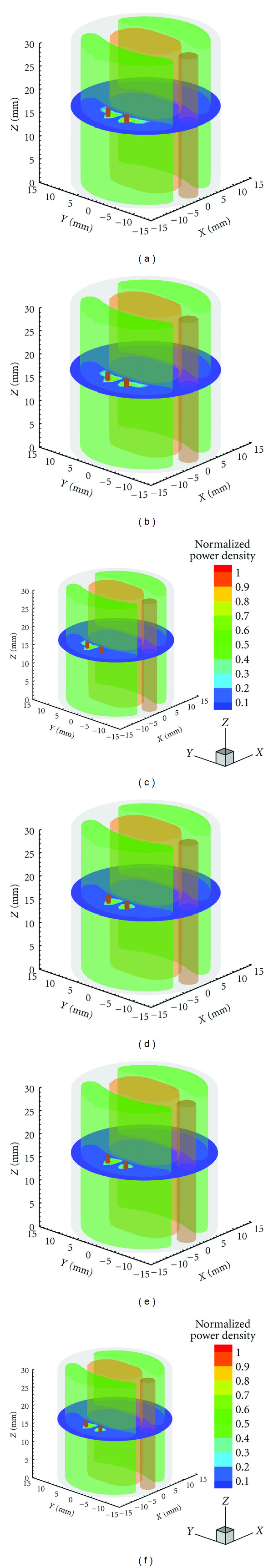
Normalized reconstruction results in phantom experiments. (a)–(c) are the results of Tikhonov + GTS with power ratio of 1 : 1, 2 : 1, and 4 : 1. (d)–(e) are the corresponding results of Tikhonov + MSDS.

The phantom model was discretized into 4202 nodes and 21721 tetrahedra. After mapping the collected optical data on the three-dimensional phantom surface, we performed four rounds of reconstruction with Tikhonov + GTS, *l*1–*ls* + GTS, Tikhonov + MSDS, and *l*1–*ls* + MSDS under different source intensity settings. The normalized reconstruction results of Tikhonov regularization method are similar to that of *l*1–*ls*. To avoid interminable description, Figure 9 only presents comparison results between Tikhonov + GTS and Tikhonov + MSDS.

For all of the testing cases considered in phantom experiments, Tikhonov + MSDS and *l*1–*ls* + MSDS can accurately detect two sources, and the maximum location error is 1.7 mm. Even for the case of real intensity ratio 4 : 1, the reconstructed source strength ratios of them were 3.12 : 1 and 2.97 : 1. In stark contrast to the proposed methods, traditional global threshold methods failed to reconstruct the weaker of the two sources, as shown in Figure 9(c). Compared with the results of using GTS (the top row of Figure 9), the proposed MSDS methods produce fewer artifacts in the reconstructed images (the bottom row of Figure 9).

## 5. Discussions and Conclusion

Accurately reconstructing and distinguishing several sources with different intensities is a challenge problem in BLT, which is also an essential ability for serial observation of disease progression or response to therapy in the same animal over time. In this work, we present a unified framework for multiple-source reconstruction by integrating a novel multiple-source detection strategy with regularization-based reconstruction process. The effectiveness of this regularization framework is validated with numerical simulations and further confirmed with phantom experiments.

The advantage of this framework is twofold. First, there is no need for *a prior* knowledge regarding source number, which is automatically estimated from the reconstruction results iteratively. Second, the regularization framework is general since it can work with different regularizers and inverse algorithms. The proposed MSDS is also easily applied to other finite-element-based reconstruction schemes to improve the final reconstruction results or image quality.

There are several limitations to the proposed method. As indicated in the experiment results, sparseness-inducing regularization method (*l*1–*ls*) performs better than *l*_2_ norm method (Tikhonov). This is mainly because *l*_1_ norm solution accords with the sparsity nature of bioluminescent source distribution in these applications. Consequently, how to select appropriate regularizer and inverse algorithm for specific BLT application is very important when using this framework.

Additionally, other regularizers can also be used in this unified framework. In fact, *l*_*p*_(0 < *p* < 1) norm regularized reconstruction has been tried for recovery of signals with weak sparsity in other image processing fields [31]. So far, related researches have not yet been reported in BLT. Based on the proposed regularization framework, our future studies will investigate the effectiveness of other forms of regularizer for the ill-posed inverse problem of BLT.

Although only the DA model is considered for the sake of simplicity, the proposed BLT reconstruction framework has no limitation on the forward model. The performance of our framework might be improved by using more accurate forward models, which is also the direction of our further work.

## Figures and Tables

**Table 1:** Optical properties of different organs.

Material	Tissue	Lung	Heart	Bone
*μ*_*a*_[cm^−1^]	0.07	0.23	0.11	0.01
*μ*_*s*_′[cm^−1^]	10.31	20.00	10.96	0.60

**Table 2:** Reconstruction results in double-source case.

Case	Power ratio	Reconstruction method	Reconstructed center and location error (mm)				Reconstructed power (nW)	
			*S*_1_^*R*^		*S*_2_^*R*^		*S*_1_^*R*^	*S*_2_^*R*^
I	1 : 1	Tikhonov + GTS	−8.95, 2.13, 14.83	1.39	−8.98, −3.57,14.73	0.28	0.65	0.28
		Tikhonov + MSDS	−8.85, 3.59, 15.14	0.22	−8.98, −3.57,14.73	0.28	0.35	0.28
		*l*1–*l*s + GTS	−8.99, 2.92, 14.77	0.62	−8.98, −3.57,14.73	0.28	0.502	0.44
		*l*1–*l*s + MSDS	−8.85, 3.59, 15.14	0.22	−8.98, −3.57,14.73	0.28	0.41	0.44

II	2 : 1	Tikhonov + GTS	−9.03, 2.69, 14.65	0.88	−8.98, −3.57,14.73	0.28	0.48	0.15
		Tikhonov + MSDS	−8.85, 3.59, 15.14	0.22	−8.98, −3.57,14.73	0.28	0.37	0.14
		*l*1–*l*s + GTS	−8.98, 2.98, 14.81	0.56	−8.98, −3.57,14.73	0.28	0.51	0.22
		*l*1–*l*s + MSDS	−8.85, 3.59, 15.14	0.22	−8.98, −3.57,14.73	0.28	0.43	0.22

III	4 : 1	Tikhonov + GTS	−9.03, 2.72, 14.66	0.85	N/A	N/A	0.49	0
		Tikhonov + MSDS	−8.85, 3.59, 15.14	0.22	−8.98, −3.57,14.73	0.28	0.38	0.07
		*l*1–*l*s + GTS	−8.97, 3.00, 14.82	0.53	N/A	N/A	0.51	0
		*l*1–*l*s + MSDS	−8.85, 3.59, 15.14	0.22	−8.98, −3.57, 14.73	0.28	0.4339	0.10

IV	8 : 1	Tikhonov + GTS	−9.03, 2.73, 14.67	0.84	N/A	N/A	0.49	0
		Tikhonov + MSDS	−8.85, 3.59, 15.14	0.22	−8.98, −3.57,14.73	0.28	0.38	0.03
		*l*1–*l*s + GTS	−8.97, 3.02, 14.83	0.51	N/A	N/A	0.51	0
		*l*1–*l*s + MSDS	−8.85, 3.59, 15.14	0.22	−8.98, −3.57,14.73	0.28	0.43	0.04
